# Commentary: The relationship between weight-adjusted-waist index and diabetic kidney disease in patients with type 2 diabetes mellitus

**DOI:** 10.3389/fendo.2024.1416865

**Published:** 2024-06-06

**Authors:** Xiao Li, Yonglong Hao, Meirong Chen

**Affiliations:** ^1^ First Clinical Medical College, Shandong University of Traditional Chinese Medicine, Jinan, China; ^2^ The Second Affiliated Hospital of Shandong University of Traditional Chinese Medicine, Jinan, China; ^3^ Department of Ophthalmology, Shandong Hospital of Traditional Chinese Medicine, Jinan, China

**Keywords:** cross-sectional studies, NHANES (National Health and Nutrition Examination Survey), weight-adjusted-waist index (WWI), DKD, diabetic kidney disease

## Introduction

We read with interest the article by wang et al. entitled “ The relationship between weight-adjusted-waist index and diabetic kidney disease in patients with type 2 diabetes mellitus” (1). In this study, the authors investigated the association between weight-adjusted waist index (WWI) and diabetic kidney disease (DKD) in the U.S. population using NHANES 2007–2018 data.WWI is a novel index for assessing obesity, which has been reported to differentiate between body fat and muscle mass and better reflect central obesity. Based on the inclusion criteria set by the authors, 5,028 individuals ultimately participated in this cross-sectional study. The authors constructed three weighted multivariate regression models to explore the relationship between WWI and DKD, with Model 1 unadjusted for covariates. Model 2 was adjusted for key demographic variables. Model 3 was adjusted for all covariates in the study. WWI was then converted from a continuous variable to a quartile categorical variable for sensitivity analyses, and subgroup analyses were performed according to factors such as gender and age to investigate the relationship between WWI and DKD in different populations. It was found that WWI was significantly and positively associated with DKD. This study innovatively explored the relationship between WWI and DKD and suggests that routine monitoring of WWI is one of the important measures to prevent and control the development of DKD. However, we still have some questions about this study.

## Adjustments for covariates

Firstly, in this study, the authors included important covariates such as age, gender, and smoking status, which is much appreciated. However, we note that in Model 3, the authors adjusted for body mass index (BMI). According to the WWI formula provided by the authors, WWI is calculated as the square root of waist circumference (centimeters) divided by weight (kilograms). In contrast, BMI is calculated by dividing weight in kilograms by height in meters. The authors did not mention whether collinearity analysis was performed, and we believe that the adjustment for BMI in Model 3 may have influenced the conclusions of this study.

## Predictive ability of WWI for DKD

Secondly, the relationship between obesity and DKD has been previously studied (2, 3), can the authors further explain the advantages of the WWI in assessing obesity or the limitations of traditional obesity assessment indicators (not limited to BMI) compared to traditional obesity assessment indicators, in order to emphasize the significance of this study. In addition, the authors assessed the predictive power through ROC analysis. For DKD risk, the corresponding AUC values were WWI index (57.19%), weight (51.23%), WC (52.23%) and BMI (49.96%). We would like the authors to compare the predictive power of WWI with other obesity assessment indicators (waist-to-height ratio, waist-to-hip ratio, etc.). The reason for this is because WWI, as a new composite index, is itself derived from weight and waist circumference by calculation. In conducting the assessment of predictive ability, we believe that comparisons should be made with other composite indices rather than with single anthropometric data such as weight and waist circumference. Thus highlighting the significance of this part of the study.

## Discussion

In conclusion, this study innovatively explored the relationship between WWI and DKD in the US population. However, we hope that the authors will take note of our questions to make this study even better and that the authors will provide additional insights into DKD in the future.

## Author contributions

XL: Writing – original draft. YH: Writing – review & editing. MC: Writing – review & editing.
